# The Effects of Static and Dynamic Loading on Biodegradable Magnesium Pins *In Vitro* and *In Vivo*

**DOI:** 10.1038/s41598-017-14836-5

**Published:** 2017-10-31

**Authors:** Youngmi Koo, Hae-Beom Lee, Zhongyun Dong, Ruben Kotoka, Jagannathan Sankar, Nan Huang, Yeoheung Yun

**Affiliations:** 1 NSF-Engineering Research Center for Revolutionizing Metallic Biomaterials, North Carolina A&T State University, Greensboro, NC 27411 USA; 2 FIT BEST Laboratory, Department of Chemical, Biological, and Bio Engineering, North Carolina A&T State University, Greensboro, NC 27411 USA; 3 College of Veterinary Medicine, Chungnam National University, Daejeon, 305-764 South Korea; 4 Internal Medicine, Hematology-Oncology Division, University of Cincinnati, Cincinnati, OH 45267 USA; 5 Key Laboratory of Advanced Technologies of Materials, Ministry of Education, School of Materials Science and Engineering, Southwest Jiaotong University, Chengdu, Sichuan 610031 PR China

## Abstract

Here we systematically assess the degradation of biodegradable magnesium pins (as-drawn pure Mg, as-cast Mg-Zn-Mn, and extruded Mg-Zn-Mn) in a bioreactor applying cyclical loading and simulated body fluid (SBF) perfusion. Cyclical mechanical loading and interstitial flow accelerated the overall corrosion rate, leading to loss of mechanical strength. When compared to the *in vivo* degradation (degradation rate, product formation, uniform or localized pitting, and stress distribution) of the same materials in mouse subcutaneous and dog tibia implant models, we demonstrate that the *in vitro* model facilitates the analysis of the complex degradation behavior of Mg-based alloys *in vivo*. This study progresses the development of a suitable *in vitro* model to examine the effects of mechanical stress and interstitial flow on biodegradable implant materials.

## Introduction

Biodegradable magnesium (Mg)-based alloys represent a new generation of biomaterials for orthopedic implants such as pins and screws^1–6^. Although these materials have been tested *in vitro*, several limitations exist in predicting *in vivo* degradation behavior^7–9^ due to differences in Mg-based alloys/purity^10,11^, testing solution composition^12–14^, immersion time^14,15^, *in vitro* testing environments^16,17^, and implant location *in vivo* ^18,19^. The use of *in vivo* modeling for initial screening and evaluation of the degradation behavior of Mg-based alloys is also hampered by high cost, long testing times, surgical effort, and ethical issues.

Therefore, developing a simplified *in vitro* model that replicates *in vivo* behavior is critical for the rapid development and clinical application of biodegradable metals. The first step in assay development is to identify the factors that most significantly affect *in vivo* degradation using a well thought-out *in vitro* test bed, taking mechanical, chemical, biological, and physiological factors into consideration^10^. A feedback-loop testing approach with *in vivo* and *in vitro* correlation will enhance our understanding of biodegradable metal degradation.

Mg-based alloys for orthopedic applications (such as pins, screws, and plates) are subject to complex dynamic stresses when they interact with human bone during fixation, especially tensile, compressive, bending, and torsion stresses^20–22^. Furthermore, dynamic loading is normally three-to-five times higher during ordinary activities such as walking and running^23^. A critical question is whether these stresses affect Mg degradation and contribute to early loss of mechanical integrity before sufficient structural bone is deposited. Hence, simulating biodegradable metal-based alloy mechanics and developing a relevant *in vitro* test bed is necessary to expedite clinical translation of new materials.

Here we report the systematic study of magnesium alloy degradation behavior with a focus on the role of mechanical stress in an interstitial flow perfusion environment. We exploit a bioreactor test bed (cyclical load/interstitial flow) that mimics the *in vivo* orthopedic device environment to understand degradation behavior. Data acquired from micro-CT images were analyzed in terms of corrosion stress cracking, fatigue corrosion, corrosion rate, uniform/localized corrosion, and corrosion product. We further characterized Mg degradation behavior using image-based finite element analysis (FEA) *in vitro* (static immersion and bioreactor-based models) and *in vivo* (mouse subcutaneous model). Finally, we systematically compared complete degradation time, effects of stress, and their correlations *in vitro* and *in vivo* (mouse subcutaneous and dog tibia implant models).

## Methods

### Biometallic pins and preparation

Three groups of Mg-based pins were used (Table S1): as-drawn pure Mg, as-cast Mg-Zn-Mn, and extruded Mg-Zn-Mn. As-drawn pure Mg (99.9%, 1.6 mm diameter) were purchased from Goodfellow (Coraopolis, PA, USA). As-cast Mg-Zn-Mn (150 mm diameter, 10 mm thick disks) and extruded Mg-Zn-Mn were obtained from Southwest Jiaotong University, China. As-cast Mg-Zn-Mn was annealed at 360°C for 8 h to ensure homogeneous distribution of the constituent metals. Extruded Mg-Zn-Mn was extruded at 330 °C into a 10 mm diameter rod^24^. Both Mg-Zn-Mn alloys were machined down to 1.7 mm diameter, and extruded Mg-Zn-Mn was cut along the extrusion direction. The entire surfaces of the Mg-based pins were polished sequentially with 400, 600, 800, 1000, and 1200 grit silicon carbide sandpaper followed by washing with isopropanol and drying in a nitrogen stream. Finally, all 5.0 mm-long Mg-based pins were embedded using epoxy resin (Epokwick^TM^, Buehler, Lake Bluff, IL) to a depth of 1 mm in 6.5 mm (OD) disc-shaped holders to leave approximately 4.0 mm of the pins exposed.

### Bioreactor test

**Figure 1:**
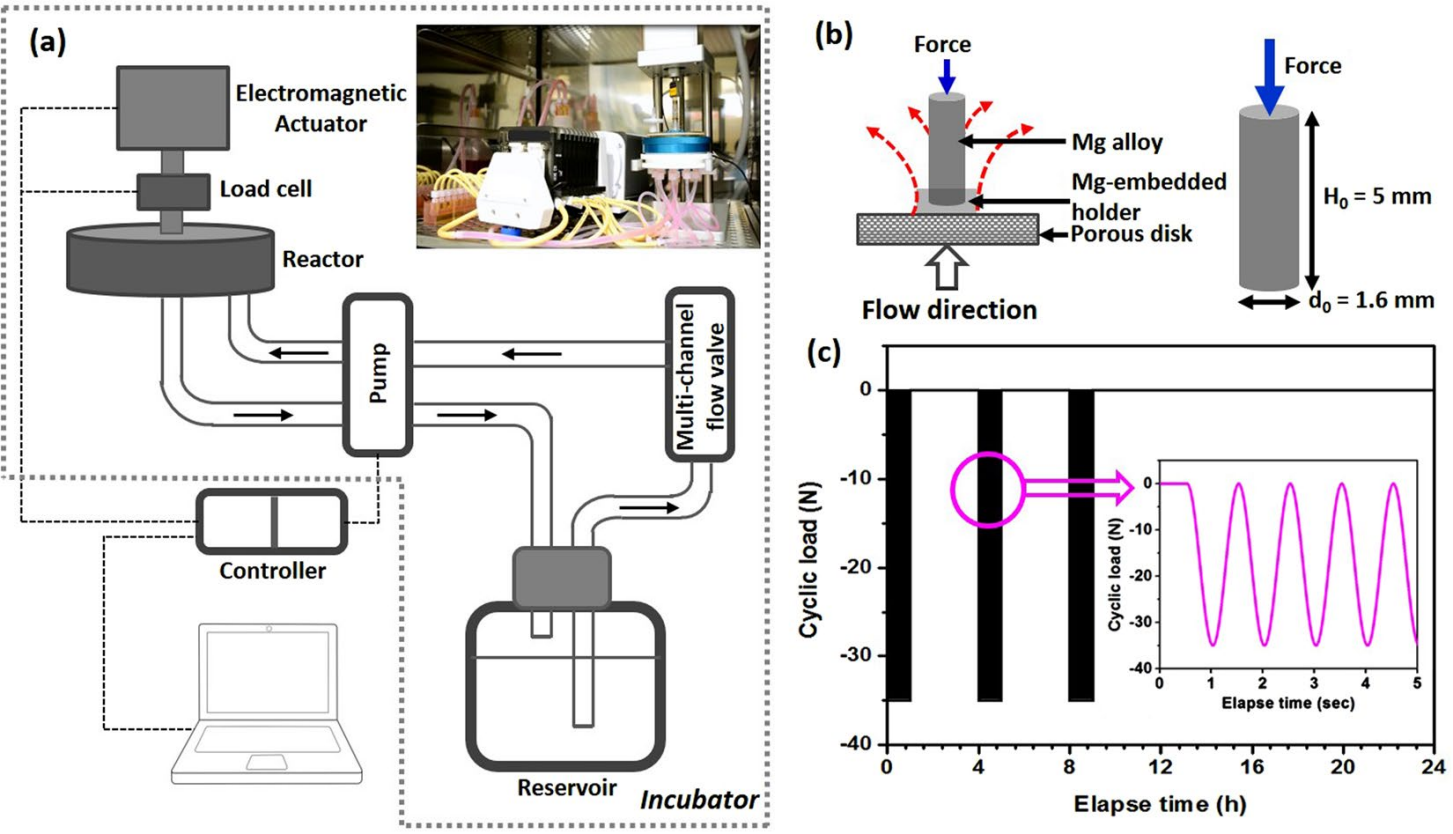
Schematic of the bioreactor for the cyclical loading/interstitial flow experiments. (**a**) Systemic bioreactor. (**b**) Inside of reactor with Mg alloy-embedded epoxy holder/porous disk and sizes. (**c**) Cyclical loading compressive force and dwell time.

A bioreactor (CartiGen C9, Instron, Norwood, MA) was used to simulate mechanical cyclical loading and flow to Mg-based pins (Fig. 1a). The bioreactor consisted of: (i) a load cell combined reactor; (ii) a pump with a multi-channel flow system; (iii) a reservoir; and (iv) a controller. Mg pins were partially mounted in epoxy resin and placed on porous disks (316LSS, 100 micron grade, 12.7 mm OD, 1.7 mm thick) (Fig. 1b). This configuration was subjected to simultaneous dynamic compressive loading and interstitial flow (Fig. 1b), making sure that the configuration did not cause crevice corrosion. All flow channels were autoclaved and filled with Dulbecco’s modified Eagle’s medium (DMEM) with 10% fetal bovine serum (FBS) and 1% penicillin-streptomycin (P/S) as a simulated body fluid (SBF). Tests were performed in a humidified incubator at 37°C in 5% CO_2_. All tests were conducted in 2 mL volumetric SBF solution per pin based on the size (matching all volumetric amounts) and volume of one well in the bioreactor reaction chamber. The SBF volume in the bioreactor reservoir was 250 mL, and the flow rate was kept at 1.5 mL/min (1.6 mm/s) (Fig. S1). Four Mg-based pins were loaded in the range 0–35 N; that is, a cyclical load of about ~ 9 N compressive force per pin was repeated three hours a day for two weeks at a frequency of 1 Hz (Fig. 1c and video S1). Mg-based pins were kept upright during all tests.

### Static immersion test

The *in vitro* static immersion test was conducted for 2, 4, and 8 weeks in 2 mL volumetric SBF solution per pin based on size (matching all volumetric amounts) and volume of the well in the bioreactor reaction chamber. All pins were kept upright during testing.

### *In vivo* murine subcutaneous test

Pathogen-free male athymic nude mice were purchased from the Jackson Laboratory (Bar Harbor, ME) and used 27 mice at 8 to 10 weeks of age. Mice were maintained in a facility approved by the American Association for Accreditation of Laboratory Animal Care (AAALAC) and in accordance with current U.S. Department of Agriculture, U.S. Department of Health and Human Services, and NIH regulations and standards. All protocols were approved by Institutional Animal Care and Use Committee (IACUC) of University of Cincinnati, and performed according to IACUC guidelines.

Corrosion of Mg-based pins was evaluated in a mouse subcutaneous model^25,26^. All samples were sterilized with 70% ethanol and UV irradiation prior to implantation. Mice were anesthetized with isoflurane through a nosecone. A subcutaneous pocket was created on the back of mice through a skin incision and nine pins each of as-drawn pure Mg, as-cast Mg-Zn-Mn, and extruded Mg-Zn-Mn (1.6 mm diameter, 5 mm length) were implanted in the subcutaneous pockets. Implant degradation was monitored for up to 12 weeks using a Kodak 4000 MM whole mouse X-ray imaging system, and pins were extracted 2, 8, and 12 weeks after implantation for *in vitro* analyses. The skin adjacent to the implants and the major internal organs (heart, lungs, liver, spleen, brain, kidneys, and intestines) were sampled, fixed in formalin, embedded in paraffin, sectioned, and stained with hematoxylin and eosin (H/E) for histological analysis.

### *In vivo* canine tibia implant test

The Chungnam National University Animal Care and Use Committee, South Korea, approved all study protocols. All protocols were performed according to IACUC guidelines. Adult beagle dogs [2–3 years old; mean body weight 9.25 kg (range, 6.5 to 11 kg)] were studied. All dogs were judged to be healthy and free of orthopedic disease based on complete physical and orthopedic examinations, complete blood count, serum biochemistry profile, and evaluation of orthogonal radiographic views of the hips, stifles, and tibiotarsal joint. As-cast and extruded Mg-Zn-Mn pins were sterilized with ethylene oxide gas prior to implantation. Dogs were premedicated with intramuscular acepromazine (0.005 mg/kg) and butorphanol (0.4 mg/kg), and 0.5% bupivacaine (1 mL/5 kg) was administered into the lumbosacral space for epidural anesthesia. After intubation, general anesthesia was maintained with isoflurane (1–2%) in oxygen (1.5 L/min). Medial and lateral skin incisions were made at the level of the distal forth of the tibia on the right and left hindlimbs. Tibias were predrilled with a 1.5 mm drill bit on the medial and lateral sides for implantation of Mg-based pins, and as-cast and extruded Mg-Zn-Mn pins (1.6 mm diameter, 5 mm in length) were inserted into these holes at random. Two orthogonal radiographic views of the tibia with their implants were taken after surgery and every four weeks until 52 weeks. All dogs were euthanized with sodium pentobarbital at 52 weeks after initial surgery. Both tibias were cut from the level of the distal third to the central tarsal bone. All bone specimens were evaluated for corrosion of Mg-based pins, integration of Mg-based pins, and host bone formation based on micro-CT.

### SEM-EDX analysis

Cross-section morphologies and element analysis of corrosion products were observed by field emission-scanning electron microscopy (FE-SEM; Hitachi 8000, Bruker, Billerica, MA) and energy dispersive X-ray spectroscopy (EDX; XFlash detector 5030 attachment on a Hitachi 8000, Bruker).

### Micro X-ray computed tomography analysis

2D or 3D images of all Mg-based pins before/after testing and after removing corrosion products were acquired by micro X-ray computed tomography (micro-CT; GE Phoenix Nanotom-M^TM^, GE Sensing & Inspection Technologies GmbH) using a voltage of 70 kV and a current of 50 μA. All images were reconstructed using the phoenix datos│x software provided with the micro-CT system. Volume ratios before/after corrosion were calculated from CT data, and corrosion rates (mm year^−1^) were calculated based on the reduction in Mg-based pin volume after corrosion from the obtained 3D data using a modification of the following equation^7^:

1 $${\rm{CR}}=\frac{{\rm{\Delta }}V}{A{\rm{t}}}$$

where CR is the corrosion rate, *∆V* is the reduction in volume equal to the remaining Mg-based pin volume after removing corrosion product, *A* is the implant surface area exposed to corrosion, and *t* is exposure time. Corrosion products were removed using chromic acid solution (200 g/L CrO_3_ and 10 g/L AgNO_3_)^27^.

## Results

### *In vitro* (bioreactor, static immersion) and *in vivo* (mouse) degradation

**Figure 2:**
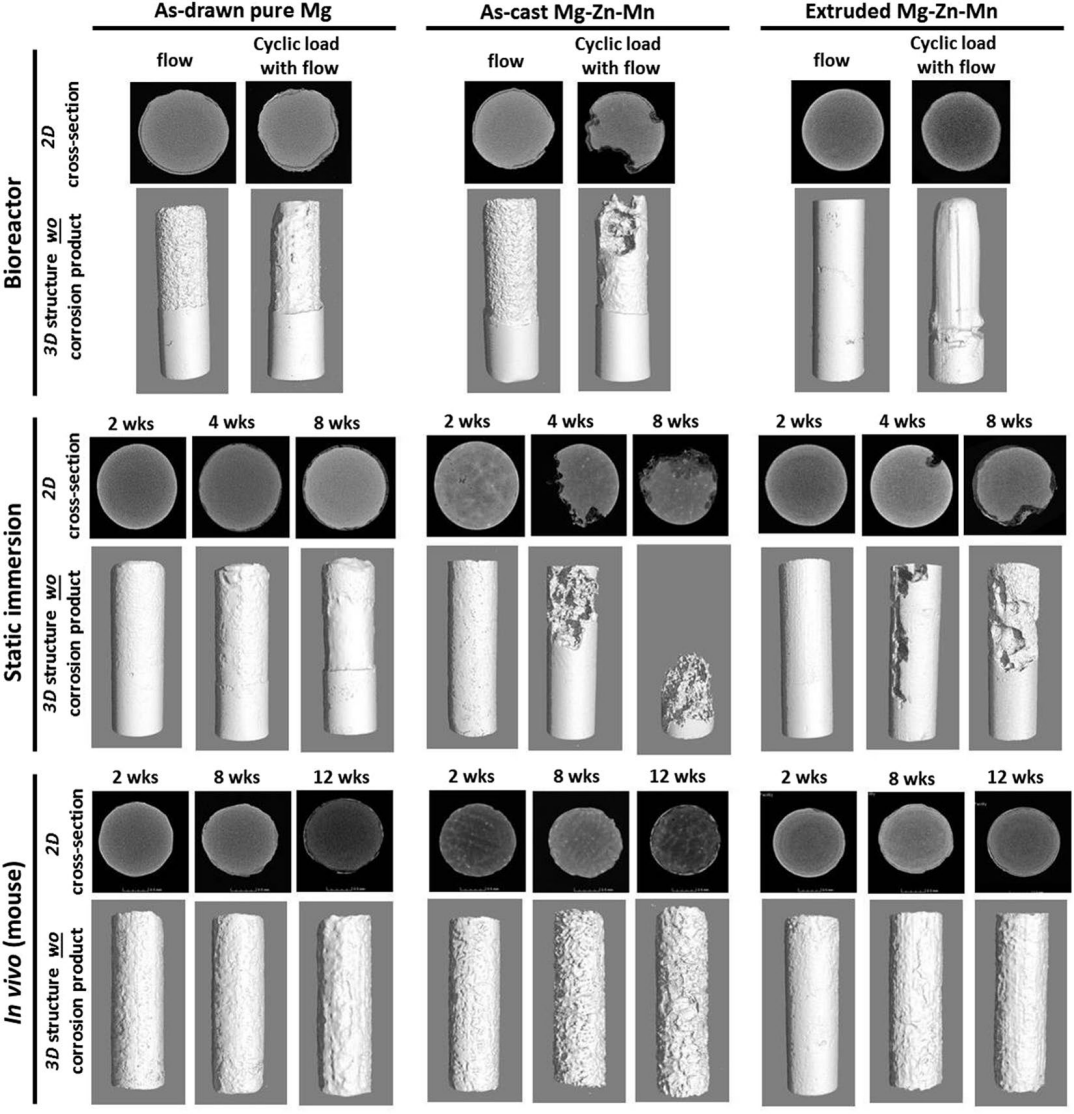
Representative cross-sectional 2D micro-CT images (with corrosion products) and 3D surface morphology images (without corrosion products) of the three different Mg-based pins (as-drawn pure Mg, as-cast Mg-Zn-Mn, and extruded Mg-Zn-Mn) in three different models. Top row: bioreactor, simulation under interstitial flow only or cyclic loading with interstitial flow for 2 weeks. Middle row: static immersion after 2, 4, and 8 weeks. Bottom row: *in vivo* (mouse subcutaneous implantation after 2, 8, and 12 weeks).

Bioreactor (two conditions: interstitial flow only and cyclical loading with interstitial flow), static immersion, and mouse subcutaneous implantation tests were performed on the three types of Mg-based pins (as-drawn pure Mg, as-cast Mg-Zn-Mn, and extruded Mg-Zn-Mn) to explore degradation behavior. 2D cross-section (before removing corrosion products) and 3D surface morphology (after removing corrosion products) micro-CT images were compared in terms of: (1) fluidic flow and (2) mechanical stress (Fig. 2). The bioreactor test revealed a wavy pattern on the pin surface under both interstitial flow only and cyclical loading with interstitial flow conditions. The degradation of all Mg pins was accelerated under these conditions, with significant pitting corrosion seen under cyclical loading with interstitial flow. Partial cracking with rapid localized corrosion was observed for as-drawn pure Mg and as-cast Mg-Zn-Mn pins. In particular, as-cast Mg-Zn-Mn pins were susceptible to localized corrosion under compressive loading conditions. Extruded Mg-Zn-Mn showed the least corrosion under interstitial flow conditions; however, uniformly localized corrosion of extruded Mg-Zn-Mn pins was increased under cyclical loading conditions. Further, the extrusion direction was visible on the surface after the corrosion product was removed. Mechanical loading might affected degradation^28^, leading to exposure of the intrinsic extrusion direction.

The Mg-based pins were immersed for 2, 4, and 8 weeks. As-drawn pure Mg pins degraded slowly and uniformly over the entire 8 weeks. However, significant localized corrosion occurred for as-cast Mg-Zn-Mn and extruded Mg-Zn-Mn after 2 weeks. As-cast Mg-Zn-Mn pins were 80 ± 2.5% degraded after 8 weeks. Even though extruded Mg-Zn-Mn pins initially degraded slowly, significant localized corrosion was still observed.

*In vivo* testing at three different time points (2, 8, and 12 weeks) revealed the highest corrosion rate, the most surface product, and a distinct wavy surface pattern for as-cast Mg pins (Fig. S2). There were no significant signs of inflammation, hemorrhage, necrosis, or discoloration in the other organs (Fig. S3). There was no significant inflammatory infiltrate in the adjacent skin, consistent with previous *in vivo* studies^25,29^.

### Degradation and tissue interface in the *in vivo* canine implant model

**Figure 3:**
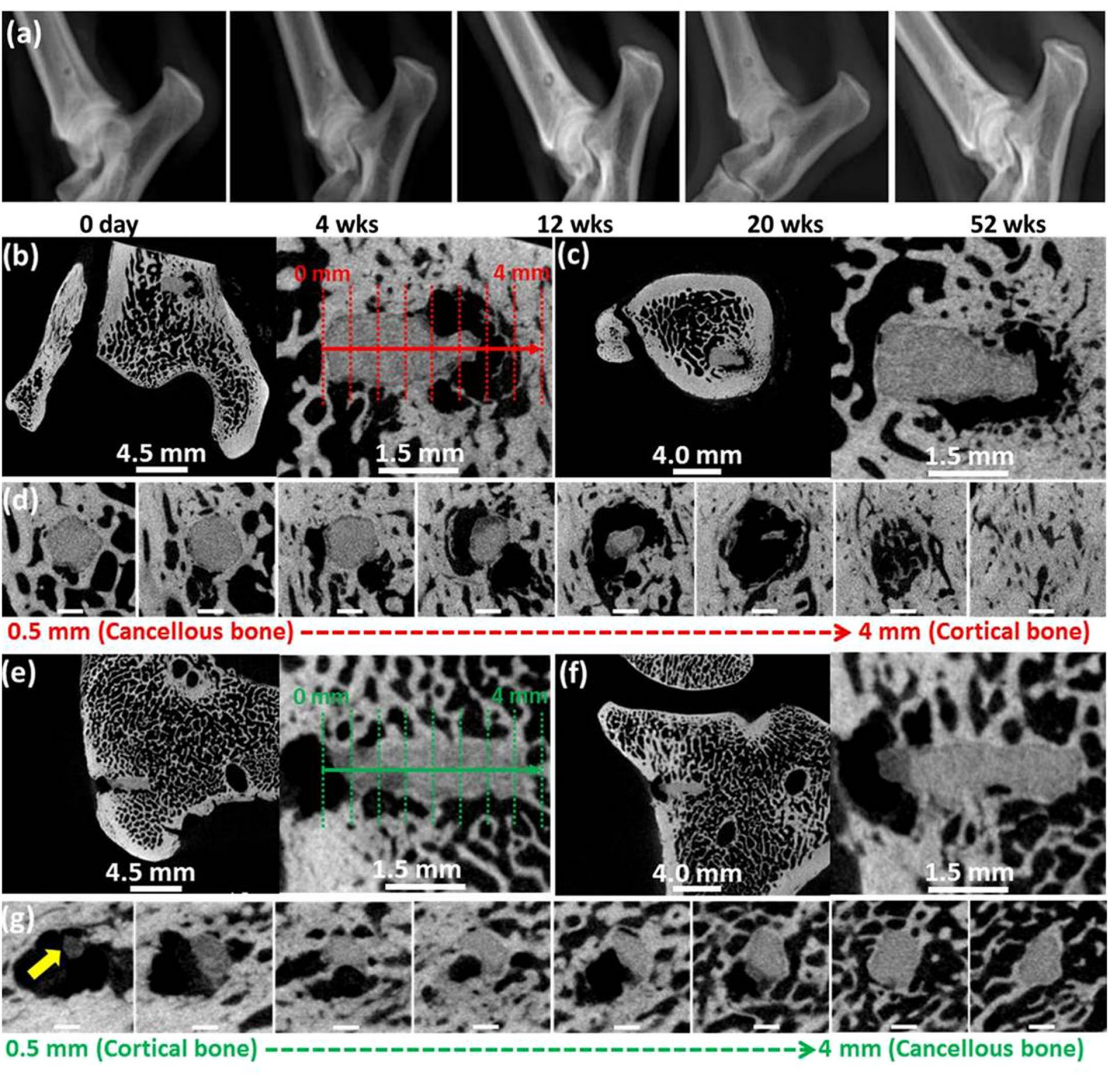
(**a**) Representative postoperative mediolateral radiographic views of as-cast Mg-Zn-Mn pins. A pin surrounded by radiolucency was observed 12-weeks postoperatively. The radiolucent area decreased in size after 20 weeks. Micro-CT images of Mg-based alloys after *in vivo* (dog) testing for 52 weeks. As-cast Mg-Zn-Mn: (**b**) front view with enlarged image; (**c**) top view with enlarged image; (**d**) right view with each 500 μm depth of (**b**). Extruded Mg-Zn-Mn: (**e**) front view with enlarged image; (**f**) top view with enlarged image; (**g**) right view with each 500 µm depth of (**e**). Scale bar of (**d**) and (**g**): 0.65 mm. Arrow in (**g**) shows the corrosion products.

X-ray images of the as-cast Mg-Zn-Mn pins were observed at different time points after implantation (Fig. 3a). The radiographic features of as-cast Mg-Zn-Mn pins and extruded Mg-Zn-Mn pins were similar. No Mg-based pins were completely degraded at 52 weeks, and no gas bubbles were observed in any Mg-based pins after 4 weeks. Mg-based pins implanted into dog tibias were analyzed by micro-CT at 52 weeks (Fig. 3b–g). As-cast Mg-Zn-Mn was mainly degraded by localized corrosion (Fig. 3b–d). Cancellous bone at the operation site had healed well in direct contact with the surrounding bone tissue with only a thin corrosion product layer [front view (Fig. 3b), top-view (Fig. 3c), and sliced views (Fig. 3d)]. In contrast, cortical bone was rapidly degraded with gas pocket formation. Extruded Mg-Zn-Mn pins were uniformly degraded along their length except at areas exposed to soft tissue (outside the bone) (Fig. 3e–g). Overall, the extruded Mg-Zn-Mn operation site had healed well in direct contact with the surrounding bone tissue and a thin corrosion product layer [front view (Fig. 3e), top-view (Fig. 3f), and sliced views (Fig. 3g)].

### Corrosion rates of the different Mg-based pins

Corrosion rates (mm year^−1^) for Mg-based alloys were ranked based on the 2-week testing periods: *in vivo* (mouse) ≈ static immersion «interstitial flow < cyclical loading with interstitial flow environment (Table 1). As-drawn pure Mg and as-cast Mg-Zn-Mn pins were more influenced by interstitial flow, while the corrosion rate of extruded Mg-Zn-Mn was more influenced by cyclical loading. As-cast Mg-Zn-Mn pins had the highest corrosion rate regardless of the *in vitro* or *in vivo* environment.Average corrosion rates were calculated based on the different testing periods *in vitro* and *in vivo* (Table S2). *In vitro*, as-drawn pure Mg and extruded Mg-Zn-Mn had similar corrosion rates to those reported in previous studies (Table S2)^10,14,30^, but as-cast Mg-Zn-Mn differed with an average corrosion rate of 1.135 mm y^−1^. Compared with the average corrosion rate *in vitro*, overall average corrosion rates *in vivo* were similar (ranges from 0.15 to 0.33 mm y^−1^). *In vivo* corrosion rates of both as-cast Mg-Zn-Mn and extruded Mg-Zn-Mn were similar in mouse (subcutaneous) and dog (tibia) models.

**Table 1:** Corrosion rates of the three different Mg-based pins tested for 2 weeks under *in vitro* (flow, cyclic load with flow and static immersion) and *in vivo* (mouse, dog^a^) conditions (mm year^−1^).

	As-drawn pure Mg	As-cast Mg-Zn-Mn	Extruded Mg-Zn-Mn
Flow	1.421 ± 0.069	1.928 ± 0.040	0.758 ± 0.189
Cyclic load/flow	1.915 ± 0.062	2.251 ± 0.288	2.113 ± 0.139
Static immersion	0.236 ± 0.022	0.527 ± 0.118	0.188 ± 0.068
*In vivo* (mouse)	0.194 ± 0.002	0.281 ± 0.002	0.167 ± 0.032
*In vivo* (dog)^a^	—	0.291 ± 0.029	0.210 ± 0.085

^a^Corrosion rates in the dog model were calculated for 52 weeks.

### Localized and uniformly localized corrosion

**Figure 4:**
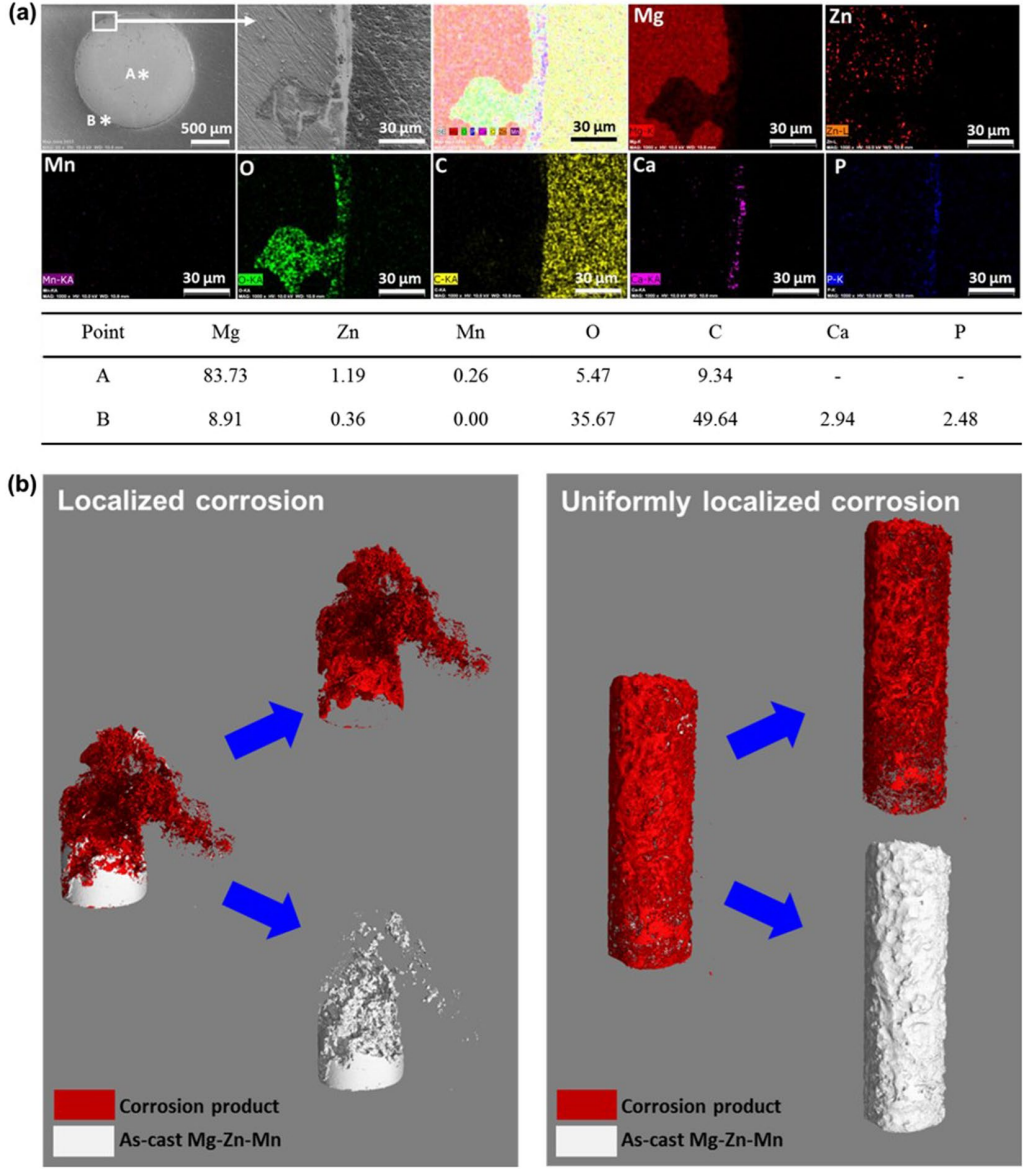
(**a**) Representative SEM and EDX analyses of as-cast Mg-Zn-Mn pins after static immersion for 2 weeks (surface morphology, mapping, and component elements by point analysis). (**b**) Representative corrosion behavior of the as-cast Mg-Zn-Mn pins. Localized corrosion of static immersion at 8 weeks and uniformly localized corrosion *in vivo* at 8 weeks.

Cross-sectional SEM and EDX analyses (Fig. 4a) of as-cast Mg-Zn-Mn after static immersion for 2 weeks showed pitting corrosion. Elements were observed in both the non-degraded and the degraded areas. Corrosion products were mainly composed of Mg, O, Ca, and P. Brucite (Mg(OH)_2_) corrosion product (Fig. S4) was located in the pitting sites of as-cast Mg-Zn-Mn, and Ca/P product formed the outermost layer of the pin. MgCO_3_·3H_2_O was only identified as a corrosion product for as-cast Mg-Zn-Mn.

This pitting corrosion after 8 weeks showed an entirely different corrosion pattern between static immersion and *in vivo* (mouse) implantation on as-cast Mg-Zn-Mn pins (Fig. 4b): (a) localized corrosion under static immersion conditions at 8 weeks; and (b) uniformly localized corrosion under *in vivo* (mouse subcutaneous) conditions at 8 weeks. Therefore, corrosion of Mg-based alloys varies according to the corrosion environment.

## Discussion

Here we show that different physiological environments (static immersion, interstitial flow, and cyclical loading with interstitial flow *in vitro* and in a mouse subcutaneous and dog tibia model *in vivo*) affected the degradation behavior of Mg-based pins. Interstitial flow and cyclical loading initially dramatically increased corrosion rate regardless of the type of Mg-based alloy (Fig. S5). Corrosion rates were accelerated four to six times by interstitial flow^3^, and cyclical loading with interstitial flow increased corrosion rates about ten times.

The increase in corrosion rate caused by interstitial flow can be explained by: (1) direct transport of fresh SBF to the implant surface; (2) deposition of Ca/P complexes; and (3) washing (possibly dissolving and detaching) corrosion products from the implant surface. In particular, interstitial flow increased the corrosion rates of as-drawn pure Mg and as-cast Mg-Zn-Mn but did not affect the corrosion rate of extruded Mg-Zn-Mn in short-term testing (2 weeks), since this latter material has a relatively fine-grain boundary (Fig. S6)^31–33^. The increase in corrosion rate caused by cyclical loading can be explained by: (1) applied stress to the Mg-based alloy-corrosion product complex; (2) detaching weakly bound corrosion product (Figs. S7); and (3) forming and propagating micro-cracks and pits (Fig. 2). Bioreactor-based testing, which simulates cyclical loading with interstitial flow, reflects the orthopedic degradation environment such as that seen in the canine model. However, bioreactors do not provide physiological osteointegration coupled with a degrading Mg surface, which might accelerate degradation overall.

The Mg-based pins implanted *in vivo* into mice (subcutaneously) and dogs (tibia) showed similar corrosion rates. Their complete degradation times according to degradation amount (%) and time (weeks) (Fig. S8 and Table S3) were estimated from the *in vitro* (static immersion) and *in vivo* (mouse subcutaneous) results: (1) about 3 months for static immersion of as-cast Mg-Zn-Mn and 6 months for extruded Mg-Zn-Mn; and (2) about 16 months (as-cast Mg-Zn-Mn) and 24 months (extruded Mg-Zn-Mn) and 16 months (as-cast Mg-Zn-Mn) and 29 months (extruded Mg-Zn-Mn) for the mouse model and dog tibia models, respectively.

In the long-term study, localized corrosion and uniformly localized corrosion were observed in *in vitro* and *in vivo* environments (Fig. 4). It is important to understand *in vivo* environments based on the screening of critical factors that influence degradation when there is tissue interaction. A further understanding of alloy microstructure in terms of surface defects/impurities, casting defects, extrusion defects, and intermetallic phase^30^ is needed since these factors greatly affect the corrosion rate, corrosion behavior, and mechanical property changes during long-term implantation. As-cast Mg-Zn-Mn showed significant localized corrosion behavior under static immersion conditions compared to the other Mg-based alloys. One reason for this might be the multiphase nature of Mg-Zn alloy^34^, since Mg_7_Zn_3_ phase can be found in the Mg-Zn-Mn alloys with >1 wt.% Zn content used in this work^35^. This second phase is known to accelerate corrosion due to microgalvanic coupling between the second phase and the alloy matrix^36^. Corrosion of as-cast Mg-Zn-Mn was initiated from small pits (Fig. 4a) and significantly increased with a discontinuous secondary phase after 2 weeks. Extruded Mg-Zn-Mn was slowly and uniformly corroded during the initial stage but localized corrosion then occurred much faster after two weeks. Due to the smaller grain size, extruded Mg-Zn-Mn was less susceptible to localized corrosion (Fig. S6)^32^. However, from the *in vivo* (mouse) degradation results, all Mg-based pins regardless of implant times showed uniformly localized corrosion (Fig. 2), and a similar surface morphology of uniformly localized corrosion was observed when Mg-based alloys were exposed to *in vivo* (subcutaneous) and *in vitro* interstitial flow conditions. However, Mg-Zn-Mn pins implanted in dog tibias degraded in a more localized way due to the mechanical loading environment (similar to the cyclical loading bioreactor test).

Cyclical loading increased degradation in terms of localized and uniformly localized corrosion for all Mg-based pins (Fig. 2). Particularly, cyclical loading under interstitial flow conditions significantly accelerated the localized corrosion of as-cast Mg-Zn-Mn, which has low mechanical strength (Fig. S6) and casting defects in the matrix^37^. This cyclical loading caused faster degradation than that of the static immersion environment. Even though extruded Mg-Zn-Mn has high mechanical strength, the corrosion rate was higher under cyclical loading with interstitial flow conditions, perhaps due to the direction of extrusion^38^. Cyclic compressive stress can lead to premature failure of Mg-based alloys through crack formation and propagation^39–41^. Shahnewaz *et al*. reported that a loss of fatigue strength in extruded Mg-2.0-Zn-0.2-Mn alloy was related to pit formation in corrosive environments^42^. If we assume that the cyclical loading bioreactor and *in vivo* dog tibia models create mechanical stimulation, then the results from as-cast Mg-Zn-Mn and extruded Mg-Zn-Mn match the degradation tendency in both models. As-cast Mg-Zn-Mn, which has a large grain boundary (Fig. S6) and lower mechanical strength (Fig. S6), were degraded, with severe localized corrosion under mechanical loading conditions. Conversely, extruded Mg-Zn-Mn has significantly smaller grain size (refined) and higher mechanical strength (Fig. S6), which led to more uniformly localized corrosion and a slow corrosion rate (cyclical loading bioreactor and *in vivo* dog tibia models). Mechanical loading critically influences localized corrosion with respect to Mg alloys.

Therefore, Mg-based pins have different mechanical strengths and microstructures that imitate different types of corrosion under specific human body conditions. Continuous interactions between the degraded surface and mechanical stress significantly affect the final integration strength of the device (Fig. S9). As-cast Mg-Zn-Mn and extruded Mg-Zn-Mn under cyclical loading with interstitial flow conditions in a bioreactor showed higher stress distribution and variation than under other conditions. However, pins implanted in the *in vivo* (mouse) model were uniformly localized corroded, and maximum stress was not affected regardless of the implantation period (Fig. S10).

In summary, we demonstrated that the bioreactor-based *in vitro* model facilitates the analysis of the complex degradation behavior of Mg-based alloys *in vivo* in terms of perfusion and mechanical loading. In particular, we found that mechanical stress, regardless of the *in vitro* or *in vitro* environment, induces stress corrosion cracking and fatigue corrosion and accelerates localized corrosion of Mg alloys with low mechanical strength and certain microstructures such as impurities, large grain boundaries, and no heat treatment^43^.

## Electronic supplementary material

Video S1 Cyclical loading

Supplementary materials
