# Supplementary material for: The Effects of Static and Dynamic Loading on Biodegradable Magnesium Pins In Vitro and In Vivo
Source: Sci Rep. 2017 Oct 31;7:14710. doi: 10.1038/s41598-017-14836-5 (PMC5665879; doi:10.1038/s41598-017-14836-5)
Supplement: Supplementary file 2 — Supplementary materials [file 41598_2017_14836_MOESM2_ESM.pdf]

Supporting Information

The Effects of Static and Dynamic Loading on Biodegradable Magnesium Pins *In Vitro* and *In Vivo*

Youngmi Koo[a,b], Hae-Beom Lee[c], Zhongyun Dong[d], Ruben Kotoka[a], Jagannathan Sankar[a], Nan Huang[e], and YeoheungYun[a,b*]

[a] NSF-Engineering Research Center for Revolutionizing Metallic Biomaterials, North Carolina A&T State University, Greensboro, NC 27411, USA

[b] FIT BEST Laboratory, Department of Chemical, Biological, and Bio Engineering, North Carolina A&T State University, Greensboro, NC 27411, USA

[c] College of Veterinary Medicine, Chungnam National University, Daejeon 305-764, South Korea

[d] Internal Medicine, Hematology-Oncology Division, University of Cincinnati, OH 45267, USA

[e] Key Laboratory of Advanced Technologies of Materials, Ministry of Education, School of Materials Science and Engineering, Southwest Jiaotong University, Chengdu, Sichuan 610031, PR China

Table S1. Chemical compositions of the three different bare Mg-based pins.

Element	Mg	Zn	Mn
As-drawn pure Mg[a]	99.9	-	-
As-cast Mg-Zn-Mn[b]	96.92	2.86	0.22
Extruded Mg-Zn-Mn[b]	97.58	2.22	0.19

[a] As-drawn pure Mg which is purchased from Goodfellow is including Al 70, Cu 20, Fe 280, Mn 170, Ni < 10, Si 50, Zn < 20 by typical analysis of company. Unit (ppm).
[b] Chemical compositions were analyzed using XRF.

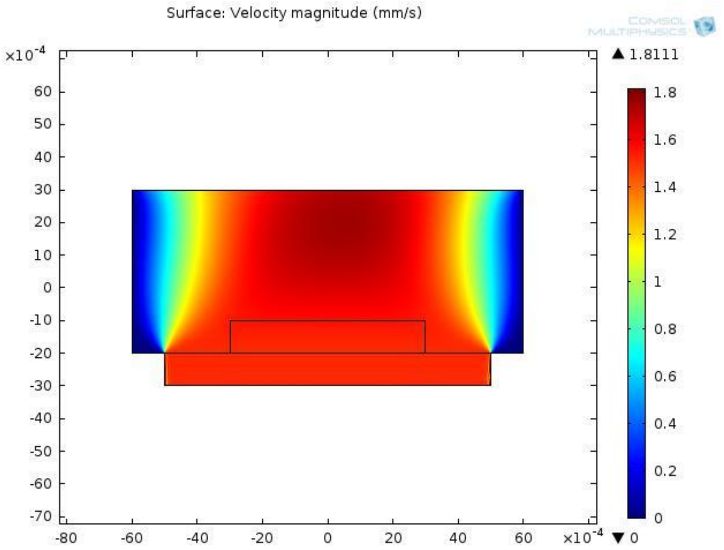

Fig. S1. Simulated fluid flow in the reaction chamber of bioreactor. Fluid flow rate in the reaction chamber of 2 mL volumetric well was simulated by COMSOL. Permeability of the porous disk that was calculated in the each well with 2 mL volumetric solution of reaction chamber in following Darcy's law; $Q = -kA(P_b-P_a)/\mu L$, where, Q is the total discharge (m^3/s), k is the product of the intrinsic permeability of the medium (m^2), A is the cross-sectional area to flow (m^2), $P_b - P_a$ is the total pressure drop (P_a), μ is the viscosity ($P_a \cdot s$), and the L is the length over which the pressure drop. Permeability of the porous disk placed in the each well with 2 mL volumetric solution of reaction chamber was kept 6.25 darcy and 1.6 mm/s during test.

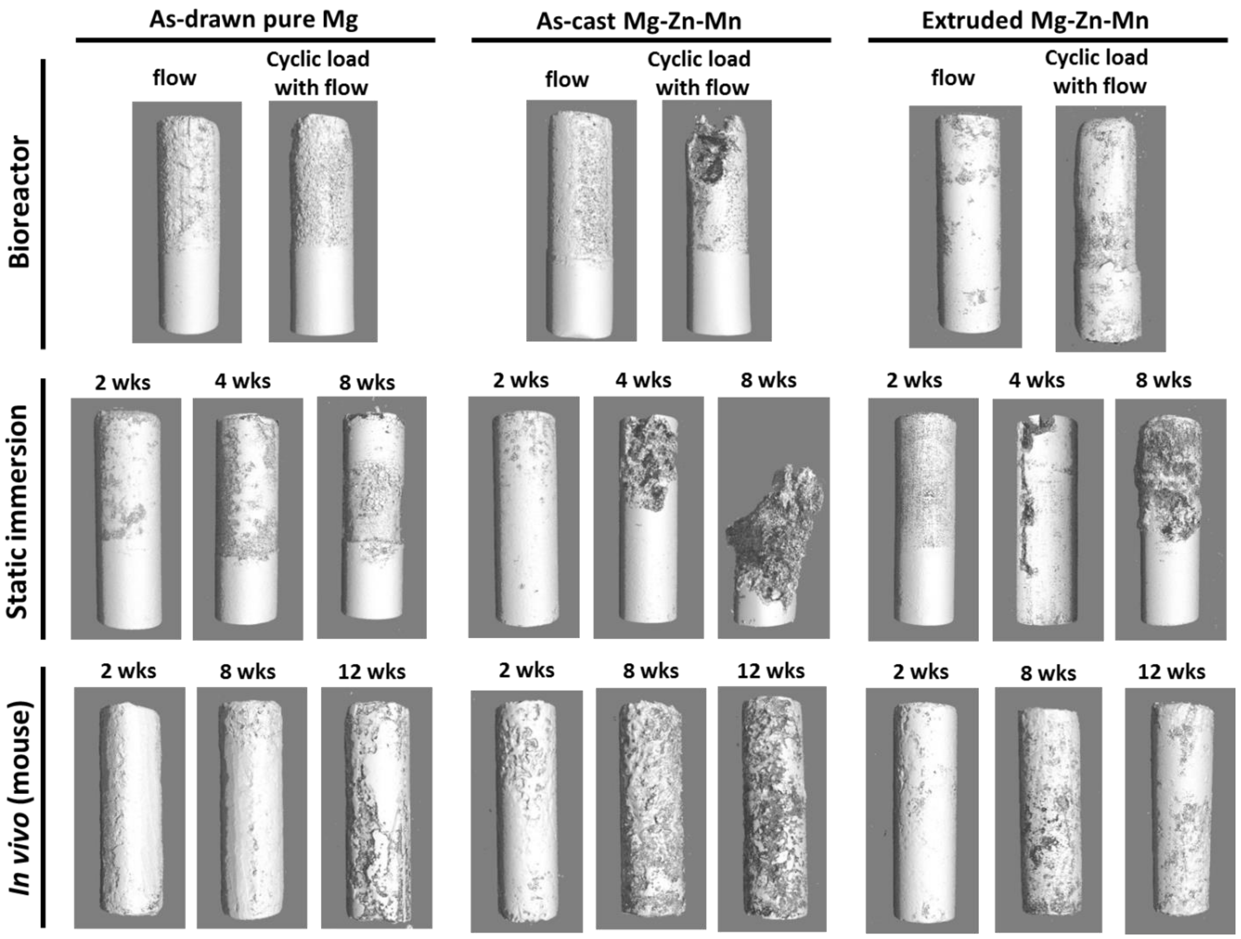

Fig S2. Reprehensive 3D surface morphology images with corrosion products of the three different Mg-based pins (As-drawn pure Mg, As-cast Mg-Zn-Mn, and Extruded Mg-Zn-Mn) at three different models.

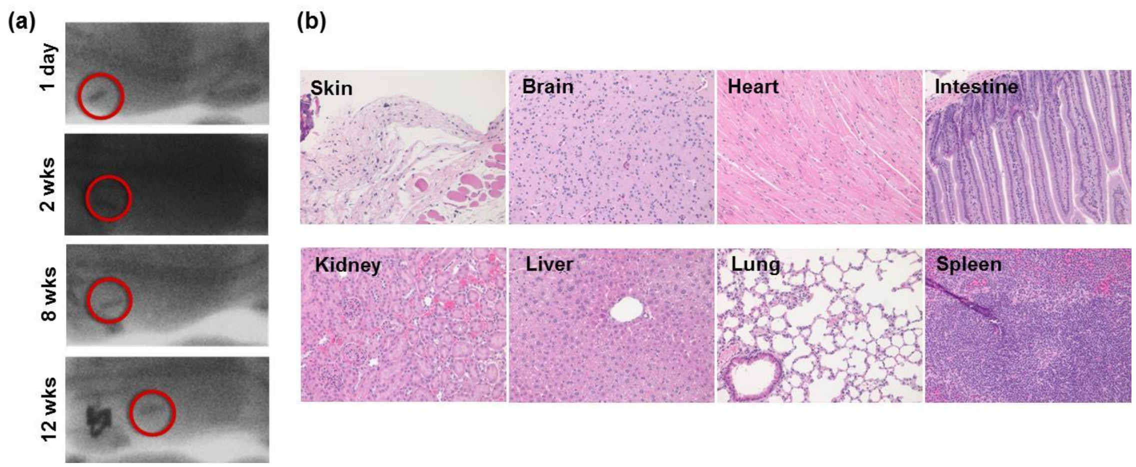

Fig. S3. Representative X-ray images and H & E staining images of as-drawn pure Mg pin, (a) As-drawn pure Mg implanted in nude mice, (b) Specific organs after 12 weeks.

Table S2. Average corrosion rates of the Mg-based pins *in vitro* (static immersion) and *in vivo* (mouse subcutaneous and dog tibia) environments and comparison with previous studies (mm year^{-1}).

	Material	Composition (wt. %)	Specified process	Testing period (weeks)	Model	Average corrosion rate (mm y^{-1})	Literature
In vitro	Pure Mg	99.9	As-drawn	2, 4, 8	Immersion (SBF)	0.317	This article
	MgZnMn	Mg-2.0Zn-0.2Mn	As-cast	2, 4, 8	Immersion (SBF)	1.135	
			Extruded	2, 4, 8	Immersion (SBF)	0.415	
	Pure Mg	99.99	NA	1, 2, 3	Immersion (EBSS)	0.474	[13]
	MgZnMn	Mg-1.0Zn-1.2Mn	NA	18	Immersion (SBF)	0.550	[10]
In vivo	Pure Mg	99.9	As-drawn	2, 8, 12	Mouse (Subcutaneous)	0.152	This article
	MgZnMn	Mg-2.0Zn-0.2Mn	As-cast	2, 8, 12	Mouse (Subcutaneous)	0.282	
				52	Dog (Tibia)	0.291	
			Extruded	2, 8, 12	Mouse (Subcutaneous)	0.186	
				52	Dog (Tibia)	0.210	
	Pure Mg	99.99	NA	1, 2, 3	Rat (Subcutaneous)	0.330	[13]
	MgZnMn	Mg-1.0Zn-1.2Mn	NA	18	Rat (Femur)	0.286	[29]

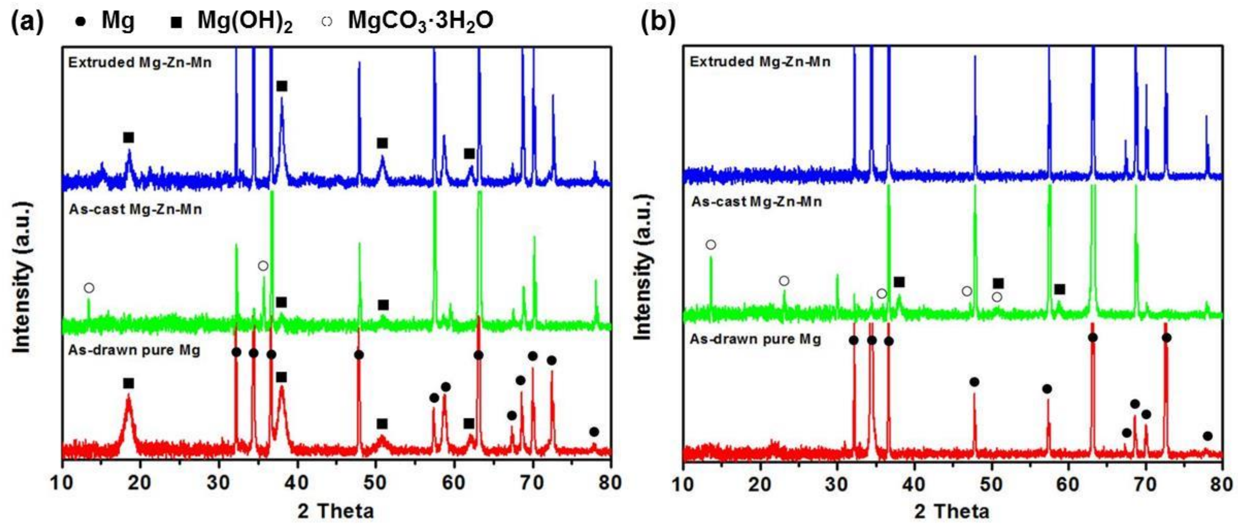

Fig. S4. XRD patterns of corrosion products of the Mg-based pins after test in the different environments. (a) Static immersion for 8 weeks, (b) Cyclical load with flow for 2 weeks.

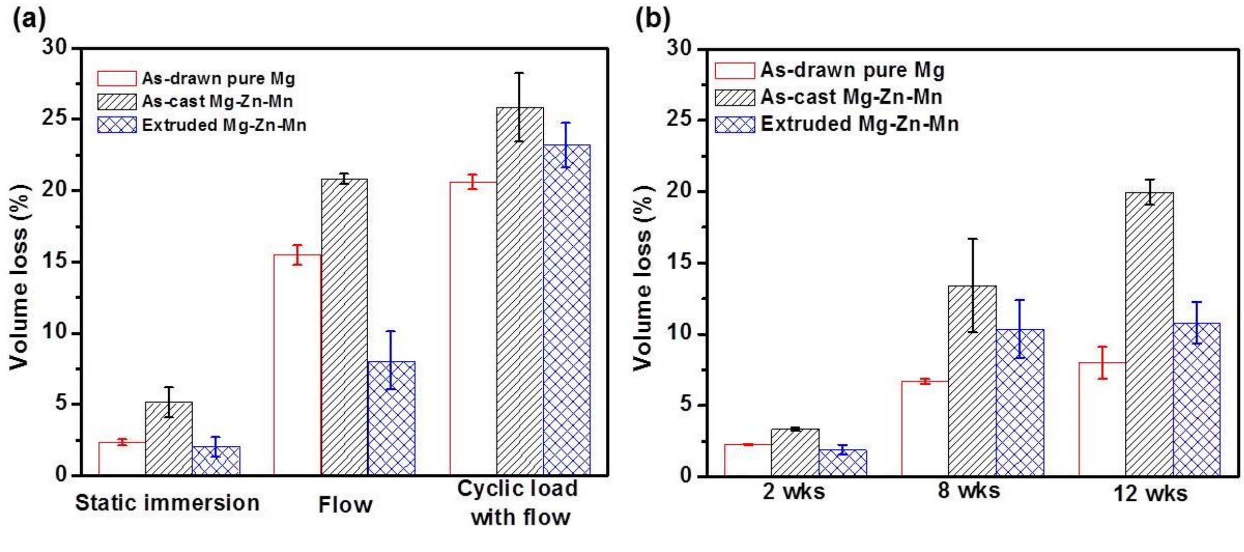

Fig. S5. Volume losses of the three different Mg-based pins after degradation test. (a) *In vitro* (static immersion, interstitial flow only, cyclic load with interstitial flow) test for 2 weeks, (b) *In vivo* test for 2, 8, and 12 weeks.

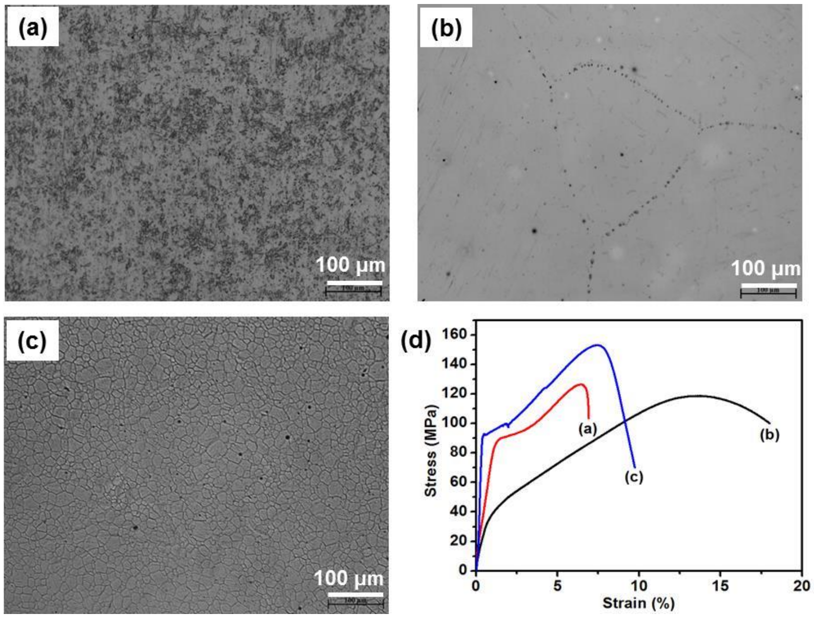

Fig. S6. Representative optical images of the microstructures and mechanical property of the Mg-based pins. (a) As-drawn pure Mg, (b) As-cast Mg-Zn-Mn, (c) Extruded Mg-Zn-Mn, and (d) Compressive stress-strain curves of the three different Mg-based pins at room temperature.

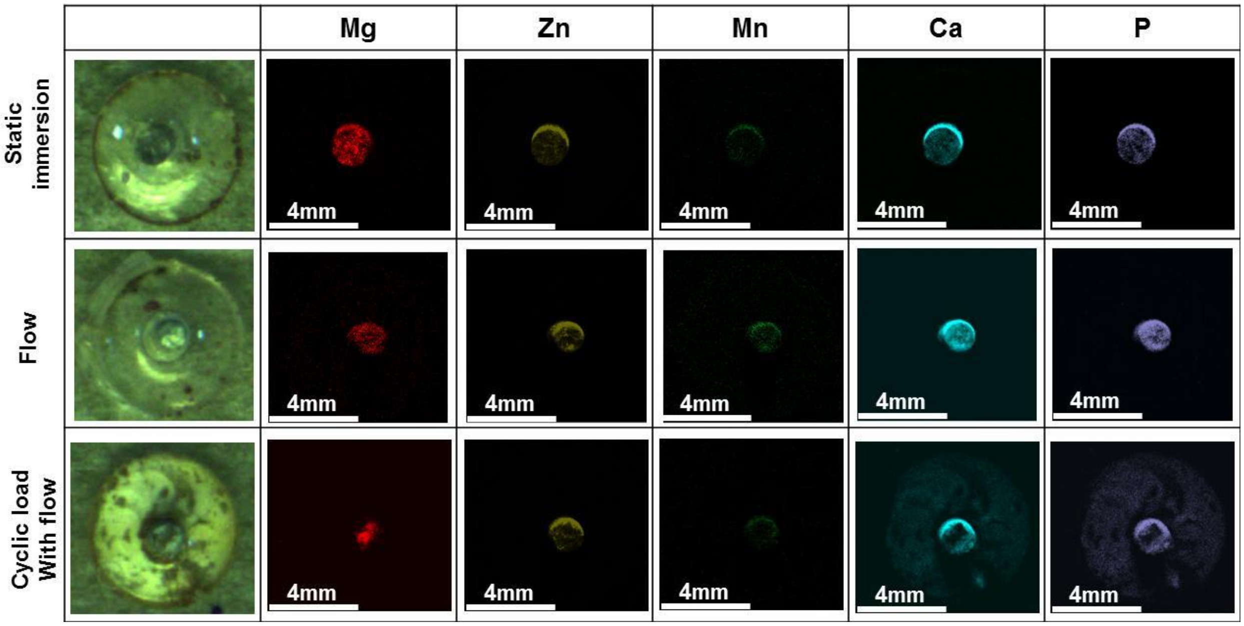

Fig. S7. Representative optical images and mapping analysis of as-cast Mg-Zn-Mn pins by XRF after test under the three different environments.

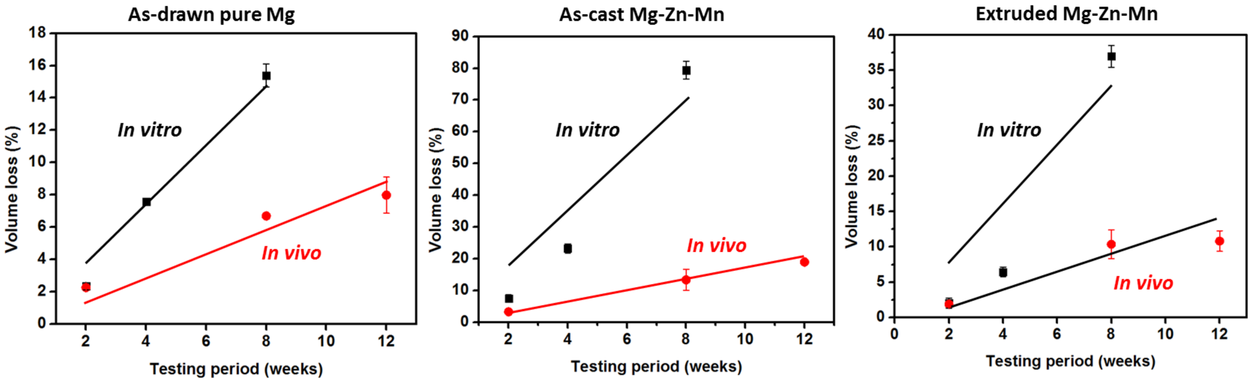

Fig. S8. Complete degradation time estimation. Linearized plots of volume loss (%) *vs*. time (weeks) of the three different Mg-based pins from *in vitro* (static immersion) and *in vivo* (mouse subcutaneous). As-drawn pure Mg: *in vitro*, y = 1.8824x and R^2 = 0.9752, *in vivo*, y = 0.7256x and R^2 = 0.8894; As-cast Mg-Zn-Mn: *in vitro*, y = 8.8514x and R^2 = 0.8865, *in vivo*, y = 1.6126.x and R^2 = 0.9969; Extruded Mg-Zn-Mn: *in vitro*, y = 3.8743x and R^2 = 0.7924, *in vivo*, y = 1.0206x and R^2 = 0.8635. Equations for estimated complete degradation time were gained from degradation volume loss *vs.* time from *in vitro* (static immersion) and *in vivo* (mouse subcutaneous) experimental results. Estimated complete degradation time of Mg-based pins in dog tibia was calculated by using scaling factors which was acquired from dividing the estimated degradation volume (result from *in vivo* mouse subcutaneous) by the actual degradation volume (result from *in vivo* dog tibia).

Table S3. Estimated complete degradation time (weeks) of the Mg-based pins *in vitro* (static immersion), *in vivo* (mouse subcutaneous), and *in vivo* (dog tibia).

	Estimated complete degradation time (weeks)		
	As-drawn pure Mg	As-cast Mg-Zn-Mn	Extruded Mg-Zn-Mn
Static immersion	53	11	26
In vivo (mouse)	138	62	98
In vivo (dog)	NA	65	117

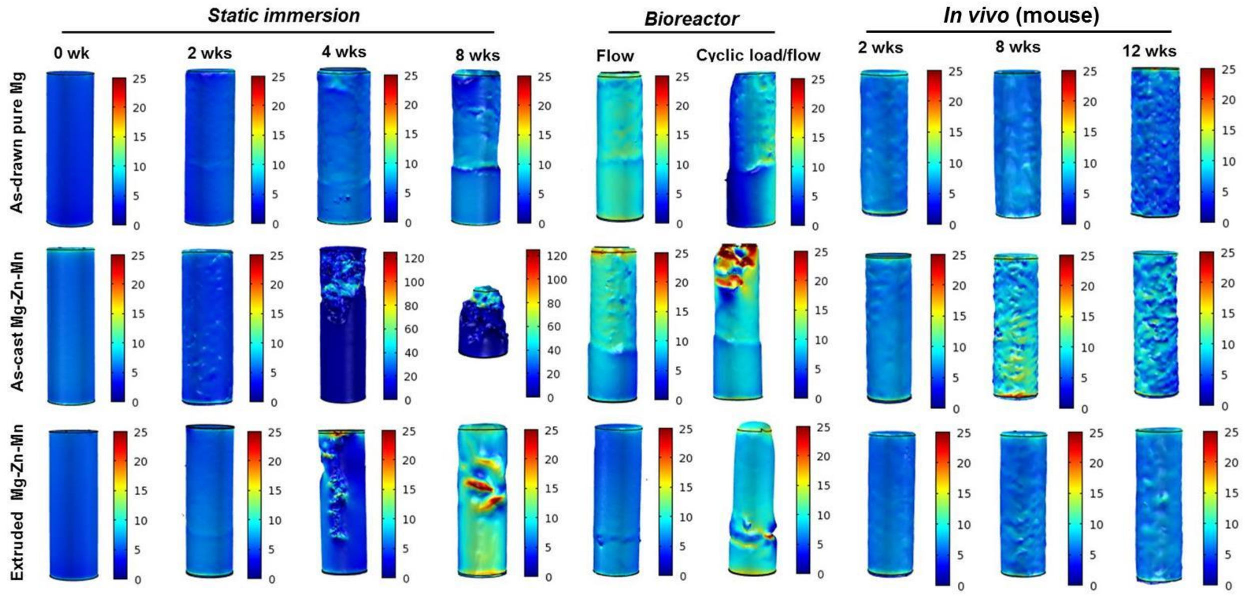

Fig. S9. Finite element analysis based on CT-images. *In vitro*: von Mises stress distributions of the three different Mg-based pins after static immersion and bioreactor test (unit: MPa). Generated by map with a color scale (lowest stress values = dark blue, highest stress values = red) on the degraded surface of the pins. *In vivo*: von Mises stress distributions of the three different Mg-based pins after *in vivo* test (unit: MPa).

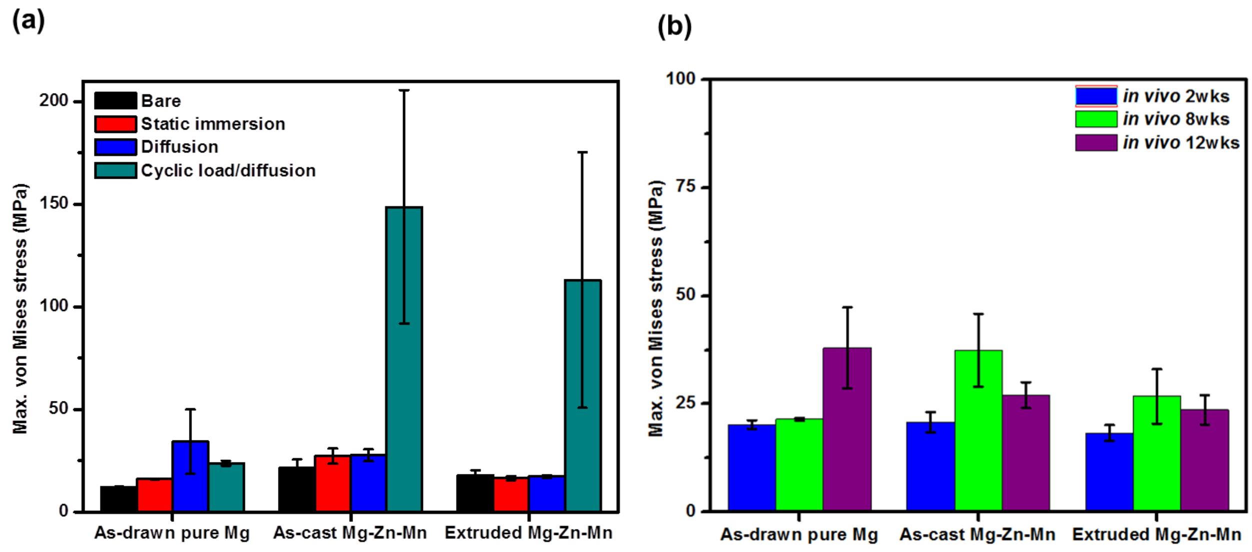

Fig. S10. Representative maximum von Mises stress of the three different Mg-based pins. (a) Bare pins and after *in vitro* test of static immersion and bioreactor for 2 weeks, and (b) After *in vivo* test.
